# Considering Heterozygous Variants in *COL4A3*, *COL4A4*, *COL4A5*: Genetic Features and Clinical Outcomes

**DOI:** 10.34067/KID.0000001344

**Published:** 2026-08-27

**Authors:** Whitney Besse, Deqiong Ma

**Affiliations:** 1 Department of Internal Medicine, Section of Nephrology, Yale School of Medicine, New Haven, Connecticut; 2 Department of Genetics, Yale School of Medicine, New Haven, Connecticut

**Keywords:** Alport syndrome, molecular genetics, genetic kidney disease

Alport syndrome manifests with hematuria, then proteinuria, and ultimately kidney failure in early to mid-adulthood, often accompanied by sensorineural hearing loss and ocular findings in X-linked Alport syndrome (XLAS) men (*COL4A5*) and autosomal recessive Alport syndrome (ARAS) patients (*COL4A3* or *COL4A4*). The majority of individuals with only heterozygous pathogenic variants in *COL4A3* or *COL4A4* have hematuria although only a small subset progress to kidney failure, and extrarenal findings are relatively rare.^1^ Despite this variability, the benefit of early recognition to allow timely initiation of renin–angiotensin–aldosterone system inhibition has led expert groups to formally adopt the autosomal dominant Alport syndrome (ADAS) nomenclature.^2,3^ ADAS is now the second most common genetic diagnosis in nephrology practice, and the field is eager to better understand the determinants of variable disease expressivity and progression to improve prognostic assessment and clinical management for these patients.

Reported kidney failure incidence in ADAS varies by ascertainment strategy. Nephrology clinic-based cohorts report ESKD in 14%–30% of patients.^4^ Population-based estimates, which consider the nearly one percent of the general population who have this genotype, suggest a kidney failure incidence of 3% or less.^3,5^ Despite the low penetrance, the ESKD risk remains substantially higher than the general population, and most individuals have at least microscopic hematuria. Accordingly, ADAS is defined as a monogenic disease with highly variable expressivity as opposed to a risk genotype.^3^

In the current issue of *Kidney360*, Gomes *et al*. characterize 62 nephrology patients from 25 families with a specific heterozygous *COL4A3* p.Gly407Arg variant followed at a tertiary nephrology center in northern Portugal.^6^ This variant is remarkably prevalent in the Portuguese nephrology population, with analysis consistent with a founder effect,^7^ while otherwise ultra rare globally (minor allele frequency 1.2×10^−6^; https://gnomad.broadinstitute.org/). Similar examples of a founder effect within the Portuguese population are described, including for kidney-relevant diseases such as Fabry Disease (*GLA* p.Phe113Leu) and transthyretin amyloidosis (*TTR* p.Val50Met). Founder populations allow for study of a sizable population sharing an identical genetic variant, reducing one source of heterogeneity while evaluating phenotype. Nonetheless, it is important to recognize that founder populations may be influenced by co-segregating genetic modifier loci and shared environmental exposures. For example, *TTR* p.Val50Met has a substantially higher penetrance in Portuguese compared with Swedish populations where the allele is thought to have arisen independently.^8^

Gomes *et al*. characterize their *COL4A3* p.Gly407Arg ADAS population using retrospective chart review supplemented with prospective interviews and auditory and ophthalmologic assessments. The median age at diagnosis of renal disease was 45 years. At the time of diagnosis, 61/62 (98%) of patients had at least microscopic hematuria, 14/62 (23%) had 0.15–0.5 g/g proteinuria, and 23/62 (37%) had >0.5 g/g proteinuria. Renal events, defined as kidney failure (eGFR <15 ml/min per 1.73 m^2^ or initiation of KRT) or a reduction of ≥30% of GFR from baseline, occurred in 16/62 (26%) patients at a median age of 67 years. Five patients (5/62, 8%) started KRT at a median age of 58±9 years. Patients with eGFR <60 and/or >500 mg/g proteinuria at study baseline had the highest likelihood of renal event. Compared with those who did not have a renal event, the 16 patients who had a renal event were diagnosed with renal disease at a later age (47.9 versus 37.1, *P* = 0.04) and thus started renin–angiotensin–aldosterone system inhibition at a later age on average (48.8 versus 43.9, *P* = 0.27) although with substantial variability in each group. This leaves open the possibility that both preexisting patient-related factors and delayed treatment influenced outcomes. Mild to moderate hearing loss was found in 13 of 38 screened (34%) and retinal changes—but without corneal or lens abnormalities more specific in Alport syndrome—were found in eight of 45 (18%) of those with ophthalmic assessment. It seems possible that some findings are attributable to age-related or alternative causes rather than ADAS itself. Overall, the renal and extrarenal outcomes in this cohort suggest that *COL4A3* p.Gly407Arg has similar consequences and breadth of phenotypic spectrum as cohorts with diverse heterozygous pathogenic variants in *COL4A3* or *COL4A4*.

Variant type has been suggested to affect disease severity or penetrance in Alport syndrome. This article's *COL4A3* p.Gly407Arg variant is a missense substitution of glycine to arginine in the collagenous domain of COL4*α*3. Glycine is uncharged and nonpolar and is the smallest of the 20 human amino acids; replacement of glycine in the Gly-X-Y repeats that define the collagenous domain of COL4 proteins (Figure 1) can have significant structural effects. Indeed, glycine substitutions are a frequent cause of nontruncating pathogenic alleles. In XLAS hemizygous men or ARAS patients, complete absence of an intact COL4*α*3, *α*4 or *α*5 protein—as would be the consequence of truncating genetic variants—results in the earliest and most inevitable kidney failure.^3^ This suggests that having an abnormal COL4*α*3/*α*4/*α*5 from a nontruncating variant may be better than nothing. In contrast, for genotypes that retain a wild-type allele in addition to the pathogenic *COL4A3/4/5* variant allele (XLAS females or ADAS patients), clinical analysis suggests that collagenous domain glycine (Gly) substitutions, particularly those introducing destabilizing amino acids (Arg, Val, Glu, Asp, Trp), result in higher penetrance of hematuria and kidney dysfunction in ADAS than truncating variants.^3^ One study suggested that Glycine substitutions in the more C-terminal exons (exons 27–52 versus exons 1–16) that disrupt earlier in trimer zippered assembly (Figure 1) resulted in earlier or more prevalent renal functional decline.^4^ Another found that individuals with heterozygous nontruncating variants outside of the collagenous domain characterized as pathogenic/likely pathogenic for ARAS had no increased incidence of Alport syndrome features than matched controls.^9^ Collectively, these findings suggest a hypothesis that the glomerular basement membrane may tolerate having only one functional allele (ADAS from truncating variant) better than having some COL4*α*3*α*4*α*5 trimers assembled with collagenous domain disruption (Figure 1).

**Figure 1:**
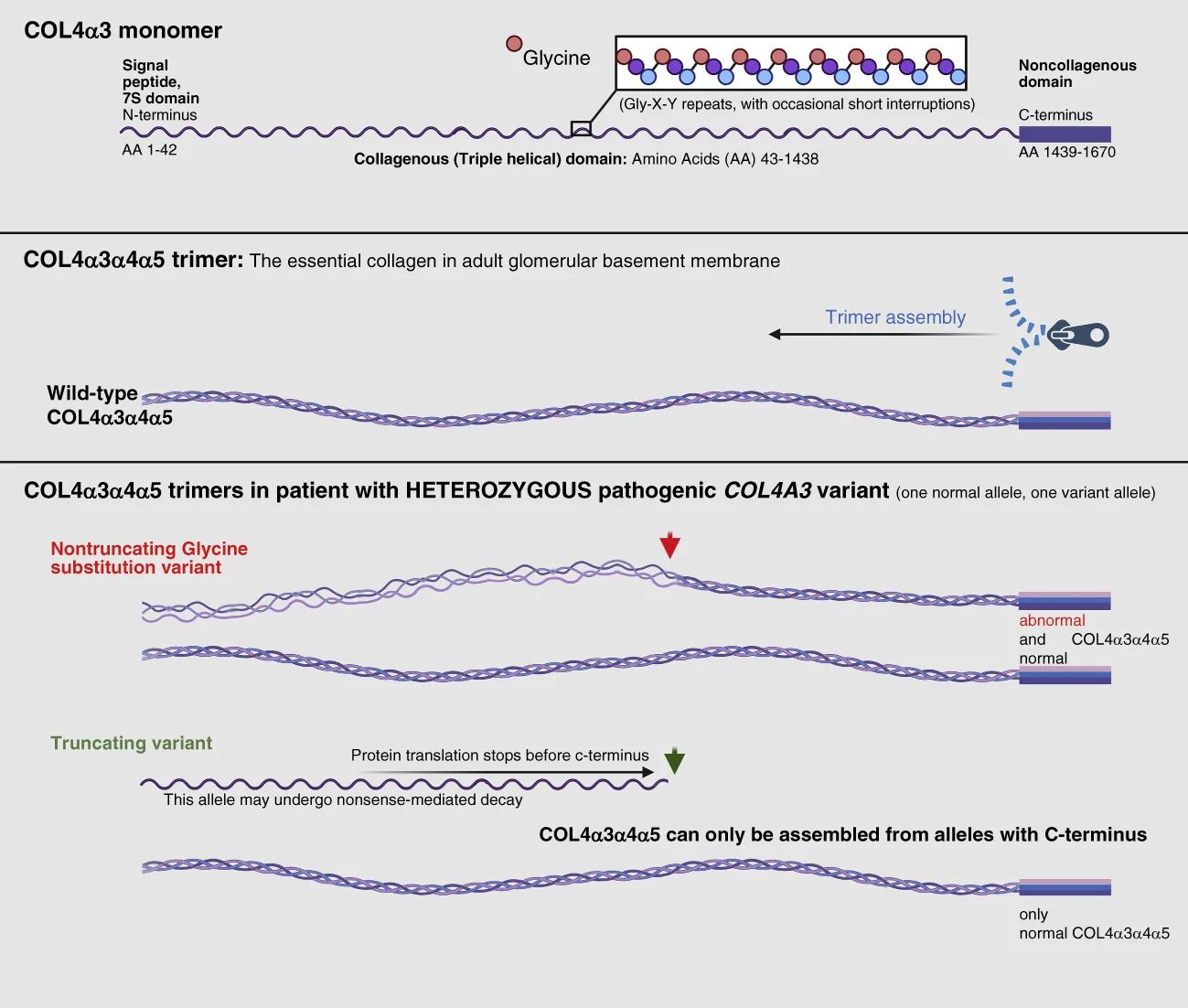
**A proposed model for higher penetrance/severity for nontruncating glycine substitutions in ADAS.** AA; amino acid; ADAS, autosomal dominant Alport syndrome.

The *COL4A5* p.Gly624Asp glycine substitution is a pathogenic allele for XLAS but with consistently milder outcomes.^3^ A proposed reason is that it lies at an interruption in the Gly-X-Y repeat. Gomes *et al*.‘s *COL4A3* p.Gly407Arg is not at such an interruption although is only one repeat away from one.

Moving beyond the disease-causing COL4 variant, a genome-wide polygenic risk score (GPS) based on >41,000 common autosomal single nucleotide polymorphisms derived for its correlation with renal function was shown to define CKD risk in individuals with monogenic kidney disease.^10^ Specifically, genetically defined ADAS individuals with low-risk GPS had similar CKD risk as the reference population (non-Alport genotypes) with intermediate-risk GPS, whereas ADAS genotype individuals with intermediate-risk or “high-risk” GPS had an odd ratios of 1.66 or 2.53 for CKD relative to the low-risk ADAS cases, respectively.^10^

In summary, clinical outcomes among individuals with ADAS likely reflect the combined effects of variant-specific properties, genetic background, environmental factors, and in some cases coexisting genetic or nongenetic causes of kidney disease. The diversity of outcomes in *COL4A3* p.Gly407Arg ADAS patients emphasizes the existence of effectors beyond the specific variant, whereas the apparent hypomorphic consequence of some glycine substitutions justifies the continued investigation of variants-specific effects. We hope that further biochemical, mechanistic, and clinical characterization will ultimately allow nephrologists to predict and maximize a benign prognosis in ADAS patients. In the meantime, recently published guidelines for the classification, diagnosis, management and treatment of Alport syndrome^2,3^ and resources from the Alport Syndrome Foundation (alportsyndrome.org) provide a framework for patient care.

## Supplementary Material
