# Supplementary material for: Considering Heterozygous Variants in COL4A3, COL4A4, COL4A5: Genetic Features and Clinical Outcomes
Source: Kidney360. 2026 Aug 27;7(8):1694–6. doi: 10.34067/KID.0000001344 (PMC13567856; doi:10.34067/KID.0000001344)
Supplement: Supplementary file 1 [file kidney360-7-1694-s001.pdf]

ASN American Society of Nephrology | JASN Journal of the American Society of Nephrology | KIDNEY360 Accessing Our World From Every Angle | CJASN Clinical Journal of the American Society of Nephrology

ASN Journal Disclosure Form

As per ASN journal policy, I have disclosed any financial relationships or commitments I have held in the past 36 months as included below. I have listed my Current Employer below to indicate there is a relationship requiring disclosure. If no relationship exists, my Current Employer is not listed.

W. Besse reports the following:
Employer: Yale School of Medicine; Research Funding: Orfonyx Bio (scientific research agreement); Yale Ventures; Patents or Royalties: Patent #WO2023230167A1 submitted/published, but not licensed to any company.; Advisory or Leadership Role: Polycystic Kidney Disease Foundation Scientific Advisory Panel; and Other Interests or Relationships: Consulting Agreement with Vertex Pharmaceuticals.

I understand that the information above will be published within the journal article, if accepted, and that failure to comply and/or to accurately and completely report the potential financial conflicts of interest could lead to the following: 1) Prior to publication, article rejection, or 2) Post-publication, sanctions ranging from, but not limited to, issuing a correction, reporting the inaccurate information to the authors' institution, banning authors from submitting work to ASN journals for varying lengths of time, and/or retraction of the published work.

Name: Whitney Besse
Manuscript ID: K360-2026-000831R1
Manuscript Title: Clinical Outcomes to be Determined: considering features of heterozygous variants in COL4A3, COL4A4, COL4A5
Date of Completion: July 23, 2026
Disclosure Updated Date: July 13, 2026

ASN American Society of Nephrology | JASN Journal of the American Society of Nephrology | KIDNEY360 Accessing Our World From Every Angle | CJASN Clinical Journal of the American Society of Nephrology

ASN Journal Disclosure Form

As per ASN journal policy, I have disclosed any financial relationships or commitments I have held in the past 36 months as included below. I have listed my Current Employer below to indicate there is a relationship requiring disclosure. If no relationship exists, my Current Employer is not listed.

D. Ma has nothing to disclose.

I understand that the information above will be published within the journal article, if accepted, and that failure to comply and/or to accurately and completely report the potential financial conflicts of interest could lead to the following: 1) Prior to publication, article rejection, or 2) Post-publication, sanctions ranging from, but not limited to, issuing a correction, reporting the inaccurate information to the authors' institution, banning authors from submitting work to ASN journals for varying lengths of time, and/or retraction of the published work.

Name: Deqiong Ma
Manuscript ID: K360-2026-000831R1
Manuscript Title: Clinical Outcomes to be Determined: considering features of single heterozygous variants in COL4A3, COL4A4, COL4A5
Date of Completion: July 14, 2026
Disclosure Updated Date: July 14, 2026
